# Household level determinants of agroforestry practices adoption in rural Legambo district of northcentral Ethiopia

**DOI:** 10.1016/j.heliyon.2025.e42765

**Published:** 2025-02-18

**Authors:** Jemal Ali Mohammed, Zinet Alye Yimam

**Affiliations:** a Department of Forestry, College of Agriculture and Natural Resources, Mekdela Amba University, Tulu Awuliya, Ethiopia; b Department of Natural Resource Management, College of Agriculture and Environmental Science, Bahir Dar University, Ethiopia

**Keywords:** Agroforestry benefits, Socio-economic variables, Logit model, Home garden, Farmers perceptions

## Abstract

The unsustainable and intensive use of land resources driven by growing populations has accelerated land degradation and reduced agricultural productivity in developing countries, particularly in Ethiopia. Agroforestry practices (AFPs) have the potential to mitigate these challenges, yet their adoption by smallholder farmers remains limited. This study aimed to assess farmer perceptions, identify existing and adopted AFPs, and analyze the socio-economic factors influencing AFPs adoption in the Legambo district of northcentral Ethiopia. A mixed-methods approach was employed, utilizing structured questionnaires for household heads (HHs) and semi-structured questionnaires for key informant (KI) interviews and focus group discussions (FGDs). The collected data were analyzed using descriptive statistics and a binary logistic regression model for quantitative analysis, while qualitative data from KIs, FGDs were qualitatively narrated. The results revealed that while farmers had positive perceptions of AFPs, adoption rates varied across practices. Scattered trees on cropland, home gardens, and boundary plantations were the most widely adopted, while alley cropping had the lowest adoption. Socio-economic factors such as gender and livestock ownership significantly promoted AFP adoption (p < 0.05), while smaller land sizes, higher education levels, and young age groups posed challenges. These findings underscore the need for targeted interventions to address barriers to adoption, focusing on enhancing access to resources and knowledge for smallholder farmers. Policymakers and development agents should prioritize tailored support for vulnerable groups to expand AFP adoption, contributing to sustainable land management and agricultural resilience.

## 1 Introduction

Land degradation is escalating globally, manifesting as indicators such as soil erosion and nutrient depletion, which pose a significant threat to the livelihoods of those in sub-Saharan Africa (SSA) [1]. Extreme weather events, such as heavy rainfall and drought, jeopardize the subsistence agriculture that supports more than half of the SSA population [2,3,4,5]. Additionally, rapid population growth has led to the expansion of cultivated lands onto vulnerable hillsides, resulting in land shrinkage and fragmentation [6,7]. Deforestation, largely driven by commercial and subsistence farming, exacerbates these issues. It is responsible for approximately 17 % of global Co_2_ emissions and continues to deplete forest resources, leading to increased land degradation, lower productivity, and heightened poverty [8,9,10,7]. In Ethiopia, the agriculture sector has struggled to mitigate soil erosion, with soil loss averaging 20 t ha^−1^ year^−1^ in the highlands [11,6,12]. This degradation incurs an annual cost equivalent to 3 % of Ethiopia's agricultural gross domestic product, worsening food insecurity and intensifying the effects of recurrent droughts [13,14].

In response to these challenges, various policies have been developed to promote agricultural productivity and address land degradation. Among these measures, agroforestry practices (AFPs) stand out. AFPs involve a strategic arrangement of elements in space and time to diversify farm and forest production while preserving natural resources [15], [16], [17]. Evidences suggests that AFPs can significantly reduce soil erosion and degradation [16,[18], [19],^20^,^21^,^22^]. International organizations, such as the world bank, international center for research in agroforestry (ICRAF), government agencies, and non-governmental organizations (NGOs), recognize AFPs' contributions to rural livelihoods [23]. Approximately 350 million AFP adopters worldwide dedicate 5–10 % of their farms to these practices [24]. Adoption of AFPs has been increasing among smallholder farmers in developing countries, especially in SSA [25,26]. AFPs offer various benefits, including enhanced crop yield, agricultural carbon emissions, improved soil fertility, environmental stabilization, and income from wood and wood products [16,27,28,29,30,7]. These benefits are crucial for low-income countries like Ethiopia, where the majority of the population relies on agriculture with minimal support from public or private sectors [16,31,19].

Despite their potential, AFP adoption has not kept pace with scientific and technological advancements [16]. Several factors, such as demographics, socio-economic status, and institutional contexts, contribute to spatial differences in AFP adoption and diffusion [32,33]. Research in Ethiopia has shown that factors like income level, education, and access to resources significantly impact AFP adoption. For example, higher income levels and educational attainment increase the likelihood of adopting AFPs due to greater awareness and financial capability [16]. Land tenure security and access to extension services are also crucial for promoting adoption [6]. Socio-economic factors such as farm size and household labor availability further influence adoption decisions [16,32]. These references collectively suggest that addressing socio-economic barriers and enhancing access to resources and knowledge are crucial for promoting AFPs among smallholder farmers in Ethiopia.

One of the regions that has experienced ongoing deforestation and land degradation is the south Wollo zone in the Ethiopian highlands, where the study area district is situated [34]. Currently, improper land management has resulted in soil erosion and severe degradation of the majority of south Wollo's highlands [32,33]. This has become one of the biggest threats to livelihoods and has further contributed to the decline in agricultural production. The problem has been made worse by recurrent drought and high rainfall variability, especially in the study area [35]. As a result, the farming systems in the Legambo district are becoming increasingly dependent on external factors and increasingly vulnerable to fluctuations in the climate. A significant portion of the district's farmers engage in subsistence farming in addition to routinely participating in safety-net programs and other humanitarian aid initiatives [28,36,37]. Since 2004, the Amhara National Regional State's (ANRS) office of agriculture has initiated and reinforced various AFPs in recognition of the issues of land degradation and deforestation being faced [33]. Smallholder farmers in the study area are still not satisfactorily adopting the practices that tackle the ongoing degradation, despite the ANRS's efforts to strengthen the AFPs. Furthermore, there has not been much focus on understanding how different socio-economic factors influence farmers' perceptions of the practices and how they are adopted [33].

While there is considerable research on agroforestry adoption globally, this study focuses on the Legambo district in north-central Ethiopia, a region with limited empirical data on AFPs adoption. This localized focus provides insights into the specific challenges and opportunities in this area. Understanding the extent and nature of AFP adoption, as well as the variables influencing adoption, is crucial for enhancing, expanding, and redesigning these practices. Therefore, this study aims to identify the factors influencing AFP adoption among smallholder farmers in the Legambo district. The specific research inquiries are.

1. What socio-economic factors affect smallholder farmers' adoption of AFPs?
2. What types of AFPs are used by smallholder farmers?
3. What are smallholder farmers' perceptions of AFPs and their benefits?

The findings will address the needs and concerns of smallholder farmers and contribute to the body of literature on AFPs adoption. Policymakers, researchers, development specialists, agricultural extension agents, and other stakeholders will find these insights valuable. The results are expected to support the implementation of improved AFPs not only in the Legambo district but also in other regions of Ethiopia and similar biophysical and socio-economic contexts.

## 2 Methodology

### 2.1 Study area

**Fig. 1:**
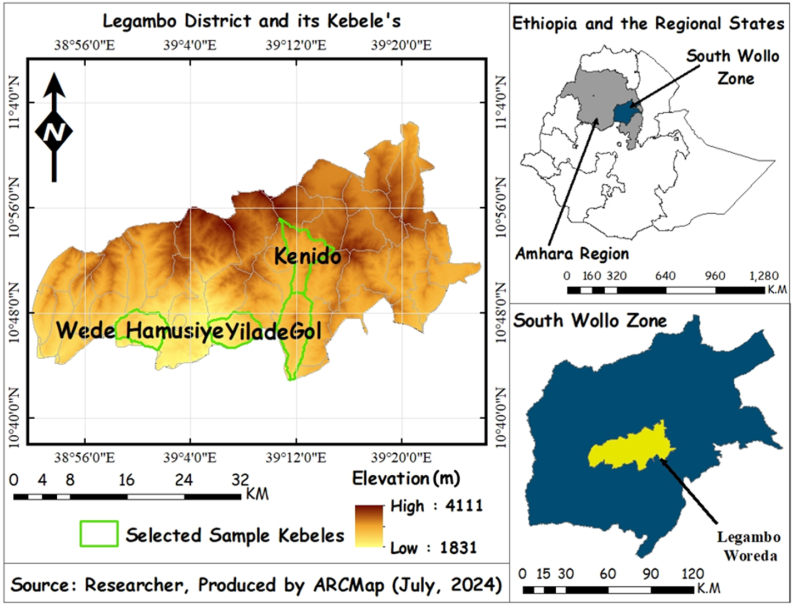
Location map of the Legambo district in Amhara region, Ethiopia.

This study was carried out in the Legambo district of northcentral Ethiopia. Geographically the district is located within the coordinates of 10°43′0′ to 11°1′00″ north and 38°54′0′ to 39°26′0″ east (Fig. 1). The district borders are as follows: Sayint to the northwest, Tenta to the north, Dessie Zuria to the northeast, Wegde to the southwest, Borena to the west, Legahida and Kelala to the south, and Were Ilu to the southeast. Its entire area is 1017.35 km^2^. Legambo district has a mean annual temperature of 14 °C and annual rainfall ranging from 768 mm in the midlands to 1393 mm in the humid highlands, with an annual average rainfall of 1037 mm. The topography of the district is characterized by a complex topography made up of plain (7 %), gentle slopes (27.4 %), mountain cliff (22.2 %) and gorges (43.3 %). An altitude of the district ranges from 1831 to 4111 m.a.s.l. 49.4 % and 48.4 % of Legambo district lays in Woyna dega (mid-land) and Dega (highland) agroecology, respectively. However, the remaining 2.2 % of the district is Wurich or high frost-prone highlands [38].

**Fig. 2:**
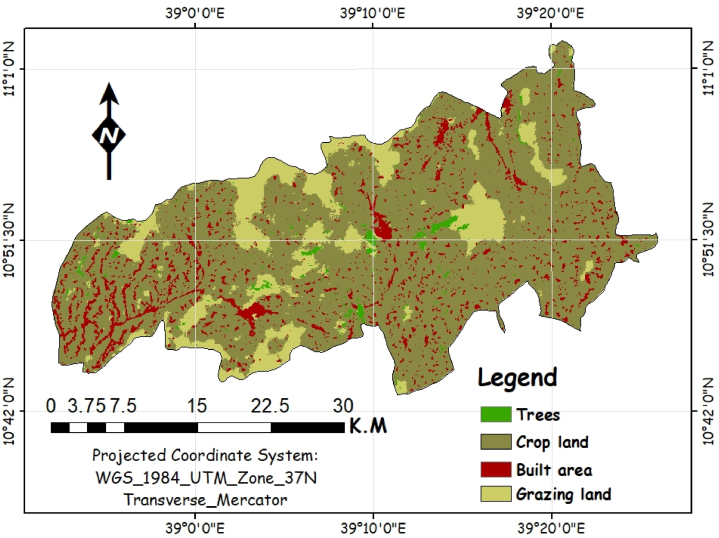
Major land use and land cover types of the study area.

The major land-use types of the district are dominated by cultivated land (69 %) (Fig. 2), grazing land (10 %), forest land (2 %), water bodies (1 %), infrastructure and settlement (3 %), and unproductive land (15 %) [38]. Moreover, Fig. 2 presents the study area's land use and land cover (LULC) for 2023, reclassified using high-resolution Sentinel-2 multispectral satellite imagery. This reclassification enables more accurate identification of various land cover types, such as agricultural, forested, and urban areas, ensuring a clearer understanding of the spatial distribution across the landscape*. Olea africana, Accacia abyssinia, Cordia africana, Eucalyptus globules, Sesbania sesban,* and other fodder tree and shrub species found in the district and they are typical tree species covering the forest land [39]. According to Ethiopian Central Statistical Agency (CSA), the district has a total population of 209,601 of whom 102,985 are males and 106,616 are females [40]. The district has a total household of 39,078 and a population density of 162.21 people/km^2^. The majority of farmers in the district rely on agriculture for their livelihoods. They are practicing ‘‘mixed’’ farming systems including crop production and sedentary livestock grazing. Crop production is mostly rain fed though in some area's irrigation is practiced [39].

### 2.2 Sampling design, techniques, and sample size

#### 2.2.1 Sampling techniques

A multi-stage sampling procedure was used in this study. First, Legambo district was chosen purposefully from south Wollo zone due to the existence of research gaps. As part of the second stage, the kebeles were stratified according to agro-ecological zones (AEZs): Dega (highland) and Woyina Dega (midland), and then two kebeles were randomly chosen from each AEZs (Table 1). The selection of two kebeles per AEZ was intended to focus on areas with distinct agro-ecological characteristics. This approach provides valuable insights into how these differences impact AFPs and allows for a detailed examination of localized factors and conditions influencing adoption rates. In addition, focusing on only two kebeles from each AEZ allows for in-depth data collection and analysis. This approach facilitates effective data gathering and processing, ensures quality control, and enables detailed assessments without compromising the study's overall validity. In the third stage, the sample HHs from each Kebeles (the smallest administrative unit in Ethiopia, similar to a neighborhood or village) were selected by a simple random sampling technique. These techniques were created for all members to have an equal chance of selection, and random selection attempts ensured the sample was representative of the whole population.

**Table 1:** Total households in each kebeles and respective numbers of sample households.

№	Sample kebeles	Agro-ecology	Number of HHs	Sample HHs	Proportion in percent
**1**	Yilada	Midland	1031	100	27.7
**2**	Wede Hamusye	Midland	919	89	24.6
**3**	Gole	Highland	676	66	18.3
**4**	Kindo	Highland	1098	106	29.4

**Total**			3724	361	100

#### 2.2.2 Sample size determination

The, sample size for this study was determined using Yamane's formula. Thus, out of the total HHs in the sampled kebeles, 361 HHs were randomly selected from both mid and highland AEZs. The number of sample HHs was determined using the following formula, which was developed by Ref. [41].

(1) $$n=\frac{Z}{1+Z{\left(e\right)}^{2}}$$

Where n is the sample size, Z is the population size (total HHs in sampled kebeles) and e is level of precision (5 %). The respective numbers of HHs were also allocated for each sampled kebeles, as shown in Table 1. The following proportional allocation formula from Ref. [42] was applied to determine the sample size at the kebele level.

(2) $$n=\frac{\mathrm{Zi}*j}{Z}$$

Where: n is the Kebele level sample size, Zi represents the total number of HHs in each sampled kebeles, j represents the overall sample size of the study based on the previously mentioned formula (Eq. (1)), and Z represents the total population size of the selected kebele.

### 2.3 Data types and sources

In this study, data from primary and secondary sources were used. The secondary data were obtained from both published and unpublished sources, including journals, articles, books, and internet sources. On the other hand, the primary data were gathered using a variety of techniques. Data were gathered from the sampled HHs on 11 important explanatory variables that were selected to influence the adoption of AFPs, farmers' perceptions of the benefits of adopting AFPs, and farmers' practices of AFPs. In accordance with earlier researchs, the following independent variables were employed in this analysis: age, gender, education, size of HH, participation in social networks, employment outside the farm, land size, economic status, livestock ownership, contact with extension agents, and conflict over land with nearby landowners (Table 2).

**Table 2:** An overview of relevant literature, hypotheses, and expected relationships between the adoption of AFPs and independent variables.

Variables	Hypothesis	Outcome	References
Age (X1)	Adopters who are older have more rights and resources, making them more likely to adopt AFPs. A young farmer, however, may find agroforestry's benefits and market response too slow. Therefore, older farmers are more likely to adopt it.	Positive	[43,36,32,44]
Gender (X2)	There are more women than men in the district of Legambo, and most decisions are made by the head of the family, who is generally a woman. Female-led family units face labor and time restrictions. Subsequently, gender (female) is expected to be negatively associated with the adoption of AFPs.	Negative	[43,45,46,47]
Level ofeducation (X3)	By reading and writing, a farmer is able to obtain and use information. Thus, AFPs adoption is expected to be positively associated with education.	Positive	[32,48,44]
HH size (X4)	The adoption of AFPs may be positively affected by a larger HHs size, with more members, and a better division of labor and better time management.	Positive	[32,47,44]
Social membership (X5)	Farmers in rural regions are supported by social networks and organizations, such as financial loans, training, information exchange, and sharing of resources. Therefore, being a member of a social network is expected to positively impact the adoption of AFPs.	Positive	[49,32,44]
Off-farm employment (X6)	In general, off-farm employment indicates a diversified income and a decrease in farm labor. Off-farm employment is expected to negatively affect AFPs adoption due to its labor and resource intensive requirements.	Negative	[32,44]
Land size (X7)	In some cases, an AFP may not be economically feasible or otherwise feasible if the farm size is below a certain threshold. Agroforestry and integrated farming may not be possible on very small plots, for instance. The adoption of agroforestry is therefore expected to be positively associated. This means the larger the farm the more the adoption.	Positive	[16,32,45,44]
Livestock (X8)	Ownership of livestock has a significant impact on the adoption of AFPs. Indicators of HHs wealth and resource availability include the number of cattle. AFPs are more likely to be adopted by wealthy farmers.	Positive	[32,37,50]
Extension contacts (X9)	The contact between a farmer and extension workers is expected to have a positive relation on adoption of different AFPs.	Positive	[51,44]
Land conflict (X10)	When farmers experience conflicts related to boundaries, it discourages farmers from taking action like adopting agroforestry. Therefore, a negative association between land conflict and adoption of agroforestry is expected.	Negative	[52,32,25]
Economic status (X11)	Agroforestry requires a lot of inputs and resources at first, and its benefits take some time to become apparent; therefore, farmers with relatively high incomes may be more likely to adopt the practices due to better access to money and labor.	Positive	[32,53,45]

Data were collected using questionnaires through interviews. Before being used for the main survey, the questionnaire underwent revisions, testing, and improvement. Closed-ended questions were utilized in HH head interviews to assess their perceptions, adoption of AFP practices, and factors influencing the adoption of AFPs. In this study, ‘adopters’ are defined as households that actively practiced at least one [33] of six AFPs: home garden, woodlot, alley cropping, boundary plantation, scattered trees on cropland, or scattered trees on grazing land. Active practice is defined as maintaining and utilizing these practices as an integral part of their agricultural systems. This includes evidence of ongoing care, such as planting, pruning, or harvesting, over a minimum duration of one year. Conversely, ‘non-adopters’ are households that have not implemented any of these AFPs. The HH heads or their spouses were chosen as respondents based on the presumption that they have satisfactory information following Ref. [32]. The questionnaire survey was carried out by the data enumerators in the farms and through direct house-to-house visits.

A five-point Likert scale was incorporated into the survey questionnaire in order to gather data on the perceptions of smallholder farmers regarding the benefits of the adoption of AFPs. According to Ref. [54], the Likert scale allows responses to be graded on a range of 1–5, 1–7, or 1–10, which enables the creation of a set of prospective measures. Numerous studies [43,55,50,44] have demonstrated the validity and reliability of the five-point Likert scale. Therefore, using a Likert-type five-point continuum scale (1 = strongly disagree (SA), 2 = disagree (DA), 3 = neutral (N), 4 = agree (AG), and 5 = strongly agree (SA)), respondents were asked to indicate the degree to which they agreed with each indicator.

In addition to the HH survey, key informant interviews (KIIs) and focus group discussions (FGDs) were conducted using semi-structured questionnaires. These tools were designed to validate the information provided by respondents and to gather additional insights on issues not raised during individual interviews. Five key informants (KIs) were selected in each sampled kebeles, with the assistance of local agricultural expert, to get general information on the criteria for assessing farmers' economic status. KIs were chosen based on their extensive knowledge of the kebeles HHs and the study area, as they had lived in the area for a considerable period and were well-acquainted with local economic conditions. Among the five KIs, three were experienced community members, one was a model farmer, and one was a local agricultural expert. All except the agricultural expert were aged above 40 years to ensure they had sufficient experience and knowledge of the local farming practices and economic conditions. The data from KIs helped to triangulate the household economic status determined by the amount of commercial crops produced and land size, following the methods outlined in Refs. [38,32].

FGDs were organized in each of the four kebeles to obtain detailed information on various aspects of AFP adoption, including challenges, benefits, and community perceptions. A total of four FGDs were conducted, one in each kebele. Each FGD included 8 participants to ensure a diverse range of perspectives. The participants were selected to represent different gender and age groups within the community, and the discussions were conducted in mixed-gender groups (both males and females participated together).

Field observations were also conducted through transect walks in each kebele, starting from the problem identification phase and continuing throughout the data collection process. Transect walks, a participatory tool, involved walking across various areas of the kebele to observe and document land use patterns, topography, forest resources, current AFP practices, and sources of livelihood. These observations, combined with KIIs and FGDs, aimed to provide a comprehensive understanding of the factors influencing AFP adoption and the socio-economic conditions in the study area.

### 2.4 Methods of data analysis

The SPSS version 26.0 was used to code and process the primary data obtained from the HH survey. The values of the variables were calculated and displayed as tables using descriptive statistics (mean, frequency, and percentage). Cross-tabulation was also computed independently for each independent variable and category (adopters and non-adopters) to compare the impact of the explanatory variables on adoption. A weighted mean was determined for each perception indicator as follows in order to analyze the data gathered using a Likert scale to evaluate smallholder farmers' perceptions of the advantages of adopting AFPs:

(3) $$\text{WM}=\frac{\left[\left(\mathrm{fSA}*5\right)+\left(\mathrm{fAG}*4\right)+\left(\mathrm{fN}*3\right)+\left(\mathrm{fDA}*2\right)+\left(\mathrm{fSD}*1\right)\right]}{n}$$

Where: weights of 5, 4, 3, 2, and 1 are assigned to perceptions of SA, A, N, DA, and SD, respectively; WM stands for weighted mean; f for frequency; and n for total sample size. Following that, the means for each indicator were categorized as follows: 1–1.49 = SD, 1.50–2.49 = DA, 2.50–3.49 = N, 3.50–4.49 = AG, and 4.50–5.00 = SA [56,44]. In the results and discussion section, the responses from the KIs, and FGDs were qualitatively narrated and briefly presented.

A binary logistic regression model (at p < 0.05) with categorical explanatory variables was used to analyze the determinant factors that affect the adoption of AFPs. Since the binary logistic regression model closely approximates the cumulative normal distribution, it was chosen for this study. From a mathematical perspective, it is simply straightforward and lends itself to a meaningful interpretation. According to Ref. [57], many farmers may also adopt multiple technologies at the same time because they complement one another and share constraints. As such, it is critical to consider the adoption of land management practices as simultaneous multiple-choice decisions [33]. In this study, we have treated the various AFPs collectively in the analysis. While we acknowledge that factors influencing the adoption of specific AFPs may vary (e.g., fodder trees versus woodlots), the decision to combine them is based on prior research, such as [33], which supports the notion that adoption can be understood in a broader context. The factors influencing AFPs adoption in this study are shared across different practices, and we have explicitly referenced this approach to justify the rationale behind combining them. This approach enhances the comprehensiveness of the analysis while ensuring that the key factors affecting adoption are adequately captured.

Thus, the general binary logistic regression model that was employed to determine AFP adoption is displayed in Eq. (4). Therefore, in order to address the study's identified problem, a number of explanatory variables related to individuals, HHs, and socio-economic status have been chosen from the literature (Table 2). An adopter or non-adopter is a categorical dichotomy, which is the dependent variable of this study. For non-adopters, the dependent variable was recorded as "0," and for adopters, it was recorded as "1.".

(4) $$\ln\left(\frac{Z}{1-Z}\right)=\beta 0+\beta 1X1+\beta 2X2+\beta 3X3+\ldots +\beta nX$$

Where: P = the probability of adopting AFPs, β0 = intercept, which is the estimation of the probability of adopting AFPs when X = 0, β1 to βn = coefficients of the independent variables representing the impact of these variables on the likelihood of adoption of AFPs, and X1-Xn are hypothesized explanatory variables.

## 3 Results

### 3.1 Socio-economic characteristics of respondents and influences on adoption of AFPs

An overview of the general demographic and socio-economic characteristics of respondents and their influence on the adoption of AFPs is presented in Table 3. The results show that the only socio-economic characteristics of the respondents that have an insignificant effect on the adoption of AFPs are conflict with neighbor landowners (P ≤ 0.053) and economic status (P ≤ 0.208). The availability of local less expensive seeds or seedlings for planting on their land could be one reason why adopters' economic has not been found to have an insignificant impact on the adoption of AFPS. Four age groups were identified from the respondent farmers: under 30 years old (8.5 %), 35–45 years old (49.5 %), 46–64 years old (37 %), and 65 years and above (5 %). The farmers who adopted AFPs are mostly in the 35–45 and 46–64 age groups. The age of farmers appears to have a significant (P ≤ 0.000) impact on the adoption of AFPs.

**Table 3:** Demographic and socioeconomic characteristics of respondents and their influence on adoption of AFPs.

Variables	Categories	Adoption of farmers		Total (%)	Chi-square	DF	P-value
		Non-adopters	Adopters				
		Count (%)					
Age	Below 30	27 (7.5)	3 (0.8)	30 (8.31)	45.40	3	0.000
	30–45	47 (13)	131 (36.3)	178 (49.3)			
	46–64	47 (13)	87 (24.1)	134 (37.12)			
	65 and above	3 (0.9)	16 (4.36)	19 (5.26)			
Gender	Male	66 (18.3)	169 (46.8)	235 (65.1)	15.99	1	0.000
	Female	62 (17.2)	64 (17.7)	126 (34.9)			
Education level	No education	94 (26)	101 (27.97)	195 (54)	110.34	4	0.000
	1-4 school	1 (0.55)	58 (16)	59 (16.34)			
	5-8 school	6 (1.66)	71 (19.6)	77 (21.33)			
	9-12 school	24 (6.64)	1 (0.27)	25 (6.93)			
	Certificate/diploma	3 (0.83)	2 (0.55)	5 (1.39)			
HH size	1–3	10 (2.8)	13 (3.6)	23 (6.37)	33.08	3	0.000
	4–6	85 (23.5)	199 (55.1)	284 (78.67)			
	7–9	28 (7.8)	8 (2.2)	36 (9.97)			
	Above 9	5 (1.4)	13 (3.6)	18 (4.99)			
Farm land	Yes	104 (28.8)	232 (64.3)	336 (93.07)	43.02	1	0.000
	No	24 (6.6)	1 (0.3)	25 (7.55)			
Source of land	Lease	27 (7.96)	16 (4.72)	43 (12.68)	28.90	2	0.000
	Freehold	19 (5.60)	88 (25.96)	107 (31.56)			
	Given by family	61 (18)	125 (34.6)	186 (51.5)			
Land size	0.125–0.5 ha.	78 (23.21)	102 (30.36)	180 (53.57)	30.4	3	0.000
	0. 51–0.75 ha.	24 (7.14)	102 (30.36)	126 (37.50)			
	0.76–1 ha.	1 (0.3)	21 (6.25)	22 (6.55)			
	Over 1 ha.	1 (0.3)	7 (2.08)	8 (2.38)			
Economic status	Poor	77 (21.33)	128 (35.46)	205 (56.8)	3.14	2	0.208
	Medium	39 (10.8)	87 (24.1)	126 (34.9)			
	Rich	13 (3.6)	17 (4.7)	30 (8.3)			
Off-farm employment	Yes	33 (9.1)	20 (5.5)	53 (14.68)	19.51	1	0.000
	No	95 (26.3)	213 (59)	308 (85.32)			
Livestock ownership	Yes	92 (25.5)	205 (56.8)	297 (82.27)	14.70	1	0.000
	No	44 (12.2)	20 (5.54)	64 (17.73)			
Extension contacts	Yes	94 (26)	219 (60.7)	313 (86.7)	30.27	1	0.000
	No	34 (9.4)	14 (3.9)	48 (13.30)			
Members to organizations	Yes	108 (29.9)	229 (63.4)	337 (93.35)	25.75	1	0.000
	No	20 (5.5)	4 (1.1)	24 (6.65)			
Conflict with neighbor land holder	Yes	3 (0.9)	23 (6.85)	26 (7.74)	3.732	1	0.053
	No	101 (30)	209 (62.2)	310 (92.26)			

The young (below 30 years) farmers were less interested in adopting AFPs (1 %) compared to the farmers under the age category of 30–45 (36.5 %). Among the total HH respondents, approximately 35 % are female, and of those, only 17.5 % have adopted AFPs. Additionally, there is evidence that AFP adoption has been significantly (P ≤ 0.000) impacted by gender. The percentage of male farmers adopting AFPs is 46.8 %, which was higher than that of female farmers (17.7 %). There are five categories for farmer education levels: no education (54 %), 1–4 school (17 %), 5–8 school (21.26 %), 9–12 school (7 %), and certificate/diploma (1.5 %). Compared to farmers who attended education under the category of 9–12, 16 % and 19.6 % of respondents who attended formal education under the categories of 1–4 and 5–8, respectively, have a high adoption rate of AFPs (Table 3).

In terms of HHs size, the majority of the adopters of AFPs had a HHs size ranging between 4 and 6 family members, which was higher than that of the non-adopters. The study area's rate of AFP adoption was found to be positively and significantly (P ≤ 0.000) impacted by the size of the HH. This is a result of the need for more forest products as a result of growing families, who require more for construction, fencing, and wood/charcoal for both commercial and domestic use. The majority of respondents in the study area (64.5 %) who adopted AFPs had farmland, and 38 % received their land as a gift from family members. As a result of these two factors (ownership of farmland and the source of that land), AFPS adoption is significantly (P ≤ 0.000) affected. The survey of this study classified the land holding size of the farmer into four categories: 0.125–0.5 ha, 0.51–0.75 ha, 0.76–1 ha, and over 1 ha (Table 3). A majority of respondents (23.21 %) who did not adopt AFPs have land sizes ranging from 0.125 to 0.5 ha, which has also significantly (P ≤ 0.000) influenced the adoption of AFPs.

Additionally, the respondents' economic status was categorized and proportionately rich (6 %), medium (59.5 %), and poor (38 %), with the majority of AFP adopters (36.5 %) falling into the medium economic status category. Since the majority of respondents (85.5 %) solely worked in agriculture, 82.5 % of them owned livestock, which had a positive and significant (P ≤ 0.000) impact on the adoption of AFPs. Similarly, 63.5 % and 60.5 % of respondents adopted AFPs indicated they were members of organizations or committees and had contacts with extension workers, respectively, showing that these two variables significantly (P ≤ 0.000) influenced adoption (Table 3).

### 3.2 Determinant factors for the adoption of AFPs

Binary logistic regression analysis showed that small-holder farmers' adoption of AFPs in Legambo district was significantly (p ≤ 0.05) influenced by age, gender, education level, farm land ownership, land size and livestock ownership (Table 4). Adoption of AFPs, however, was positively correlated with gender (P ≤ 0.000), farm land ownership (P ≤ 0.002) and livestock ownership (P ≤ 0.000). According to the odds ratio value, farmers who own farmland adopt AFPs at a rate that is over 53 times higher than that of their counterparts. The adoption of AFPs by farmers was also found to be positively and significantly (P ≤ 0.000) correlated with livestock ownership. Conversely, the results of the binary logistic regression model indicated that the independent variables of age, education level, and land size had a significant negative impact on the dependent variable, which is the adoption or non-adoption of AFPs (Table 4). Reducing the age of the HH head resulted in a significant (P ≤ 0.04) decrease in AFP adoption. The adoption of AFPs by HHs was found to be negatively and significantly (P ≤ 0.043) influenced by the farmers' educational level. It is generally accepted that farmers will be better able to understand and implement AFPs if they have more educational attainment. This research, however, showed that the adoption of AFPs contradicted this general assertion. Adoption of AFPs in the study area was also found to be negatively and significantly (P ≤ 0.02) influenced by the total land size of the HH (Table 4). The adoption rate of the practice is approximately three times lower among landholders with comparatively smaller land areas (0.125–0.5 ha) compared to those with larger land areas. It was also noted that during the experts and FGDs, five obstacles to adopting AFP were identified. These obstacles include land size and scarcity, laziness, a shortage of tree seeds or seedlings, the issue of the survival rate of tree seedlings, and overgrazing/deforestation.

**Table 4:** Binary logistic regression showing the influence of demographic and socioeconomic factors on adoption of AFPs.

Variable	Categories	B	S.E.	Wald	DF	Sig.	Exp (B)
Age	Below 30	−5.88	2.861	4.225	1	0.04a	0.003
	30–45	−0.446	1.863	0.057	1	0.811	0.640
	46–64	−0.312	2.034	0.024	1	0.878	0.732
	65 and above (Ref.)						
Gender	Male	1.41	0.314	20.295	1	0.000a	4.110
	Female (Ref.)						
Education level	No education	0.477	0.924	0.267	1	0.605	1.612
	1-4 school	4.466	1.360	10.777	1	0.001a	87.000
	5-8 school	2.876	1.007	8.159	1	0.004a	17.750
	9-12 school	−2.77	1.369	4.100	1	0.043a	0.062
	Certificate/diploma (Ref.)						
HH size	1–3	−1.399	1.043	1.800	1	0.180	0.247
	4–6	−1.139	0.882	1.670	1	0.196	0.320
	7–9	−0.580	1.197	0.234	1	0.628	0.560
	Above 9 (Ref.)						
Farm land ownership	Yes	3.980	1.027	15.009	1	0.002a	53.538
	No (Ref.)						
Source of land	Lease	−1.967	0.591	11.070	1	0.001	0.140
	Freehold	0.743	0.428	3.011	1	0.083	2.102
	Given by family (Ref.)						
Total land size	0.125–0.5 ha.	−3.19	1.371	5.419	1	0.020a	0.041
	0. 51–0.75 ha.	0.112	1.354	0.007	1	0.934	1.118
	0.76–1 ha.	1.166	1.967	0.351	1	0.553	3.208
	Over 1 ha. (Ref.)						
Economic status	Poor	0.684	1.118	0.374	1	0.541	1.982
	Medium	0.084	1.112	0.006	1	0.939	1.088
	Rich (Ref.)						
Off-farm employment	Yes	−0.445	2.076	0.046	1	0.830	0.641
	No (Ref.)						
Livestock ownership	Yes	1.58	1.867	0.098	1	0.000a	4.558
	No (Ref.)						
Contact with extensions	Yes	0.120	0.798	0.022	1	0.881	1.127
	No (Ref.)						
Membership to organizations	Yes	1.110	1.153	0.927	1	0.336	3.036
	No (Ref.)						
Conflict with neighbor land holder	Yes	−0.72	1.056	22.545	1	0.754	5.55
	No (Ref.)						
Constant		−0.831	2.057	0.163	1	0.686	0.436

Statistically significant (P = ≤0.05) influence on the adoption of AFPs is indicated by letter a.

### 3.3 Description of AFPs in the study area

The most popular AFPs were scattered trees in croplands and woodlots, which were planted by 63.7 % and 58.2 % of smallholder farmers, respectively. The third most adopted AFP was scattered trees grown in rangelands (56.8 %) established for different purposes, including fuelwood and forage. The fourth most adopted AFP was home garden, which was adopted by 55.7 % of respondents (Table 5). The nature of home garden AFP, which can be practiced in any homestead, does not require a large amount of land, involves all family members in its management, and is protected from free-grazing may be the reason why 55.7 % of respondents adopted the practice. Alley-cropping (13.6 %) was less adopted due to the fact that certain plant species, seeds, or plant materials are required for the establishment.

**Table 5:** AFP types practiced by smallholder farmers in the study area.

Statements	Frequency (%)	
	No	Yes
Do you have scattered trees on cropland (scattered trees grown in crop lands)?	131 (36.3)	230 (63.7)
Do you have wood lots (plot of trees planted for benefits like fuel, fodder, timber etc.)?	160 (44.3)	201 (55.7)
Do you have trees on rangelands (scattered trees grown in range lands)?	156 (43.2)	205 (56.8)
Do you practice home garden AFPs (trees/shrubs with annual crop and livestock's)?	151 (41.8)	210 (58.2)
Do you have boundary plantation/trees planted in single or multiple line for boundary?	193 (53.5)	168 (46.5)
Do you practice alley-cropping AFPs (cultivated crops between row of trees)?	312 (86.4)	49 (13.6)

### 3.4 Farmer's perception on the benefits of adopting AFPs

A number of statements about the advantages of adopting AFPs were given to the respondent farmers, who were asked to rank them from strongly disagreeing to strongly agreeing. Most respondents agreed with the set of adopting AFPs benefit statements, with a small minority strongly agreeing and disagreeing (Table 6). For example, the statement "adoption of AFPs conserves soil and water" received a total mean score of 3.85, indicating strong agreement (5.3 %), agreement (84 %), and disagreement (9 %) among the sample of respondents. Additionally, AFPs enhance soil nutrients and boost farm income; 79.2 and 76.7 % of respondents agreed with these statements, respectively, and the total mean fell between 3.5 and 4.5, suggesting that a significant portion of respondents agreed with the statement.

**Table 6:** Perception of the respondent on the benefits of adopting AFPs.

Statements	Respondents' response						
	SA (5)	AG (4)	N (3)	DA (2)	SDA (1)	Mean	SDE
	Count (%)						
Adoption of AFPs conserves soil and water	19 (5.3)	303 (83.9)	6 (1.7)	33 (9.1)	0 (0)	3.85	0.644
Adoption of AFPs improves soil nutrients	0 (0)	286 (79.2)	22 (6.1)	53 (14.7)	0 (0)	3.65	0.724
Adoption of AFPs increases farm income	0 (0)	277 (76.7)	17 (4.7)	67 (18.6)	0 (0)	3.58	0.785
AFPs can improves soil cover	0 (0)	276 (76.5)	26 (7.2)	59 (16.3)	0 (0)	3.60	0.754
Adoption of AFPs improves micro-climate	0 (0)	275 (76.2)	6 (1.7)	80 (22.2)	0 (0)	3.54	0.833
Adoption of AFPs improves crop production	17 (4.7)	280 (77.5)	21 (5.8)	45 (12.5)	0 (0)	3.75	0.747
AFP reduces fuel wood gathering time	11 (3)	271 (75.1)	24 (6.6)	55 (15.2)	0 (0)	3.66	0.769
AFPs practices have an economic advantage	15 (4.1)	261 (72.2)	40 (11)	45 (12.5)	0 (0)	3.81	0.718
AFPs reduced risk of complete crop failure	10 (2.8)	234 (64.8)	20 (5.5)	97 (26.9)	0 (0)	3.43	0.917

Average						3.65	

Conversely, some farmer respondents indicated that they were unsure or did not believe that implementing AFPs was necessary to achieve the set of AFP benefit statements, thus they answered in the neutral category of the statements that were provided. The respondents' perceived percentage of disagrees with each of the benefits of AFPs ranged from 9 to 27 %. Only 9 % of sample respondents disagreed with the benefit statement, which stated that using AFPs would conserve soil and water. These findings indicated how crucial it was for farmers to contact extension workers, as this contact significantly (p ≤ 0.018) influenced how farmers perceived the contribution of AFPs to soil and water conservation (Table 6). On the other hand, the largest percentage of farmers' perceptions that fell under the disagree category (27 %) supported the idea that adopting AFPs would not benefit them by reducing the risk of complete crop failure. The respondents' mean perceptions were likewise generally positive and above average for each statement of benefits of adopting AFPs, as indicated by the mean (3.43–3.85) and overall average scores (3.65) (Table 6).

The advantages of AFPs as perceived by HH respondents were also examined using cross-tabulation and percentage analysis concerning various explanatory variables (Table 7). The findings showed that age, education level, land ownership, and extension contacts were independent variables that had a significant impact on HHs' perceptions of the advantages of adopting AFPs. The age of respondents had a significant (p ≤ 0.000) impact on the respondents' perceptions of the benefits of adopting AFPs, with increasing age being associated with increased perceptions. Furthermore, in comparison to those who did not attend formal education, the respondents perceived the adoption of AFPs positively in light of their education, which had a significant influence (p ≤ 0.000). While the respondent's perception was positively impacted by education, the adoption of AFPs appears to have been negatively impacted by education level for the reasons covered in section 4.2. Ownership of farmland has also had a significant (p ≤ 0.018) impact on respondents' perceptions. With only 10 % of HH respondents disagreeing, the majority of respondents (70.4) acknowledged and agreed with the benefits of adopting AFPs. Furthermore, the contacts HH respondents had with extension workers had a significant (p ≤ 0.018) impact on farmers' perceptions (71.5 %) regarding the benefits of adopting AFPs. The cross-tabulation result (Table 7) generally showed that the selected socio-economic factors had both significant and non-significant influences on the dependent variable, which were farmers' perceptions of the benefits of adopting AFPs.

**Table 7:** Influences of selected variables on respondents' perceptions of adopting AFPs.

Variables	Categories	Perception of farmers					Chi-square	P-value
		SA	AG	N	DA	SDA		
		Count (%)						
Age	Below 30	1 (0.3)	20 (5.5)	0 (0)	9 (2.5)	0 (0)	30.75	0.000a
	30–45	0 (0)	143 (39.6)	27 (7.5)	8 (2.2)	0 (0)		
	46–64	0 (0)	109 (30.2)	20 (5.5)	5 (1.4)	0 (0)		
	65 and above	0 (0)	15 (4.2)	1 (0.3)	3 (0.83)	0 (0)		
Gender	Male	1 (0.3)	175 (48.5)	33 (9.1)	26 (7.20)	0 (0)	2.798	0.424
	Female	0 (0)	103 (28.5)	13 (3.6)	10 (2.77)	0 (0)		
Education level	No education	0 (0)	134 (37.1)	28 (7.8)	33 (9.14)	0 (0)	55.104	0.000a
	1-4 school	0 (0)	56 (15.5)	1 (0.3)	2 (0.55)	0 (0)		
	5-8 school	0 (0)	59 (13.3)	17 (4.7)	1 (0.3)	0 (0)		
	9-12 school	1 (0.3)	24 (6.6)	0 (0)	0 (0)	0 (0)		
	Certificate/diploma	0 (0)	5 (1.4)	0 (0)	0 (0)	0 (0)		
Farm land	Yes	0 (0)	254 (70.4)	46 (12.7)	36 (9.97)	0 (0)	20.8	0.000a
	No	1 (0.3)	24 (6.6)	0 (0)	0 (0)	0 (0)		
Land size	0.125–0.5 ha.	0 (0)	137 (38)	29 (8)	14 (3.88)	0 (0)	6.327	0.388
	0. 51–0.75 ha.	0 (0)	98 (27.1)	13 (3.6)	15 (4.16)	0 (0)		
	0.76–1 ha.	1 (0.3)	16 (4.4)	3 (0.8)	2 (0.55)	0 (0)		
	Over 1 ha.	1 (0.3)	4 (1.1)	1 (0.3)	2 (0.55)	0 (0)		
Off-farm employment	Yes	1 (0.3)	42 (11.6)	4 (1.1)	6 (1.66)	0 (0)	7.281	0.063
	No	0 (0)	236 (65.4)	42 (11.6)	30 (8.31)	0 (0)		
Livestock ownership	Yes	0 (0)	226 (62.6)	41 (11.4)	30 (8.31)	0 (0)	6.334	0.096
	No	1 (0.3)	52 (14.4)	5 (1.4)	6 (1.66)	0 (0)		
Extension contacts	Yes	0 (0)	258 (71.5)	44 (12.2)	11 (3)	0 (0)	10.01	0.018a
	No	1 (0.3)	30 (8.3)	2 (0.6)	15 (4.15)	0 (0)		
Members to organization	Yes	1 (0.3)	256 (70.9)	46 (12.7)	34 (9.42)	0 (0)	4.134	0.064
	No	0 (0)	22 (6.1)	0 (0)	2 (0.6)	0 (0)		
Adoption	Adopter	1 (0.3)	182 (50.4)	17 (4.7)	14 (3.88)	0 (0)	2.155	0.541
	Non-adopter	0 (0.3)	96 (26.6)	29 (8)	22 (6.09)	0 (0)		

Statistically significant (P = ≤0.05) influence on the adoption of AFPs is indicated by letter a.

## 4 Discussion

In this study, younger farmers demonstrated less AFPs compared to those aged 30–45 years. This could be attributed to the longer time needed for AFP adoption, which may discourage younger, less experienced farmers. Additionally, young individuals often seek employment opportunities in urban areas, which could further reduce their interest in adopting AFPs in rural settings. In contrast, elderly farmers are more likely to stay year-round in their rural residences, making them more inclined to engage in agroforestry activities [33].

The study also revealed that male-headed HHs were significantly more likely to adopt AFPs compared to female-headed HHs. This is likely due to the historical control of land and other resources by men in SSA [47]. Women often face additional barriers, such as limited access to labor for land preparation and the physical demands of AFPs, which can be more labor-intensive than other agricultural technologies 58. This aligns with previous findings from south Wollo zone [33] and Jama district [32], which found that male-headed households had higher adoption rates for AFPs, though the positive impact of gender was not always statistically significant.

Furthermore, ownership of livestock was a strong predictor of AFP adoption, with livestock owners adopting AFPs at a rate more than four times higher than those without livestock. This is likely because livestock owners seek additional feed sources for their animals, driving them to adopt AFPs. A similar trend was observed with larger livestock holdings, where farmers with more livestock were more inclined to adopt AFPs. This finding is consistent with previous studies [32], which highlight the role of livestock as a key factor in encouraging AFP adoption.

The result also confirmed that older farmers were more likely to adopt AFPs than younger farmers. This result is consistent with studies that indicate that age positively influences the adoption of agricultural technologies, as older farmers generally have more experience with complex technologies like agroforestry [52,59,60,32,47]. Older individuals often have a better understanding of the long-term benefits and management requirements of AFPs, which require more time and experience to fully implement. Research conducted in the Gobu Seyo district of East Wollega by Ref. [61] has also demonstrated a positive correlation between the adoption of coffee shade agroforestry technologies and the number of years of agricultural experience.

Interestingly, the study found that the adoption of AFPs was negatively correlated with education levels. Respondents with higher education adopted AFPs at a rate three times lower than those with little to no formal education. This is in contrast to other studies that found education to have a positive influence on AFP adoption [52,59,60,62]. A plausible explanation is that highly educated individuals may be more likely to pursue non-agricultural employment, which often offers more immediate financial returns. Additionally, formal education often overlooks practical agricultural knowledge, which may reduce the inclination to adopt AFPs.

Another factor influencing adoption was land size. Farmers with larger landholdings were more likely to adopt AFPs, while small-scale farmers were often reluctant to do so due to the perceived risk of losing valuable land to tree planting. This finding is supported by research indicating that larger landowners are more willing to invest in agroforestry, while small-scale farmers are often constrained by limited land resources [32,47]. It is essential for stakeholders to consider land size when designing agroforestry interventions, offering guidance on how to incorporate AFPs without jeopardizing smallholders' essential land. Therefore, it is imperative to assist farmers in planning how to incorporate AFPs on land that they believe to be small in comparison to their other uses. The land holdings of the farmers must also be taken into account by stakeholders when designing AFPs. In terms of barriers to adoption, the FGDs highlighted that seedling survival rates were a major challenge. This was consistent with other studies that identified low seedling survival as a significant barrier to AFP adoption [63,32]. Extension services should focus on providing farmers with knowledge and techniques to improve seedling survival rates and manage AFPs more effectively. In addition [63,32], reported that the adoption of AFPs was negatively impacted by land size, scarcity, and low seedling survival rates. These findings are consistent with the findings of the current study. In addition, the findings corroborated [64] result that the two primary obstacles to AFP adoption in Pakistan's Indus River Basin are farmer laziness and deforestation.

The greater adoption of AFPs in the study area was largely driven by the benefits gained from practices like scattered trees and woodlots. The widespread use of scattered trees in croplands may have resulted from farmers realizing the effects of current soil erosion and the beneficial role that scattered trees play in reducing erosion and enhancing soil fertility. Additionally, the widespread adoption of woodlots can be attributed to the low cost of establishment and ease of handling through coppicing after harvesting 65, whereas the lower adoption of alley-cropping may be due to the specific plant species, seeds, or plant materials required for its establishment. They require additional management, especially to prevent cattle from ingesting or trampled plants until they reach their ideal size. This aligns with other studies, like the one carried out by Ref. [33], who discovered that the lack of robust local legislation to address the problem of free grazing and camel damage to planted seedlings intended for alley cropping contributed to farmers in two (Harbu and Dessie Zuria) districts of south Wollo zone having a low adaptation rate for alley-cropping AFPs.

Farmers' perceptions of the benefits of adopting AFPs are generally positive, as confirmed by feedback from FGD members and KIs. They claim that planting a variety of leguminous trees in the farm field improves yields by decreasing soil erosion and increasing soil fertility, which is also consistent with [48] findings. These results are also in line with those of [66,9,44], who found that, in comparison to no AFPs, AFPs help conserve soil and water, improve soil nutrients, and increase the income of agroforestry adopters. Conversely, 27 % of the respondents supported the idea that adopting AFPs would not benefit them by reducing the risk of complete crop failure. According to KIs, there are plausible reasons for these conclusions: farmers believed that since the canopy of trees covered a large portion of the farm field, crop yields were lower because light could not enter; this caused crop failure rather than rising it. This suggested that the respondents did not know enough about the different agroforestry management practices. The rationale for this result was consistent with that of [44], who discovered that farmers in their study area were less aware of the advantages of AFPs in terms of lowering the risk of total crop failure due to the shade effect of AFPs that was already existed. The respondents' mean perceptions suggests that farmers had a positive perception of the advantages of adopting AFPs. In spite of this, some farmers who were aware of the benefits of AFPs did not adopt them. It was socio-economic factors, rather than awareness, that influenced adoption of AFPs, as indicated in section 3.2. Similar to this result [67], showed that bio-physical and socio-economic factors affect adoption of AFPs, rather than awareness and perception. On the other hand, farmers' perceptions of the statements regarding the benefits of adopting agroforestry, namely that AFPs can lower the risk of total crop failure, were on average 3.43. The implication here is that the farmers were not entirely convinced of AFPs' ability to lower the likelihood of total agricultural failure.

The results also indicated that farmers who interact with extension agents have greater awareness and are better equipped to adopt AFPs, with more positive perceptions, as agroforestry is a knowledge-based practice [32]. Interestingly, being an adopter or non-adopter of AFPs did not seem to have any significant impact on the respondent's perception. On the other hand, farmers who had a positive attitude towards the roles of AFPs were more likely to adopt them. This finding is consistent with other studies that have shown a positive association between HH perceptions of the value of adopting AFPs and their adoption, such as those by Refs. [32,68]. However, the generalizability of the study's findings may be limited by the sample size and regional representation, which may not reflect broader populations or other areas with different socio-economic or environmental contexts. Additionally, the reliance on self-reported data from surveys and interviews introduces potential biases or inaccuracies related to perceptions and adoption practices. The study represents a specific moment in time, and variations in socio-economic conditions or agricultural practices over time could influence AFP adoption rates differently. External factors such as regional policy changes or climate fluctuations, which were beyond the study's control, might also impact AFP adoption and perception in ways not fully addressed by the study. These limitations should be taken into account when interpreting the results and considering their relevance to other contexts.

## 5 Conclusions

Based on the findings, this study addressed three key research questions related to AFPs adoption among smallholder farmers. Firstly, several socio-economic factors were found to significantly influence the adoption of AFPs, with gender, farm land ownership, and livestock ownership showing a positive impact. Other factors such as age, education, and land size had mixed effects. Despite the perceived benefits of AFPs, barriers such as land scarcity, seedling survival issues, and overgrazing were identified as hindrances to wider adoption. Secondly, the types of AFPs most commonly practiced included scattered trees in woodlots (55.7 %), home gardens (58.2 %), range lands (56.8 %), and cropland (63.7 %), while alley-cropping was the least adopted method. These practices provided economic advantages, soil protection, and environmental benefits to the farmers. Thirdly, farmers generally had a positive perception of AFPs, acknowledging their role in meeting basic needs and improving sustainability. However, challenges like tree seedling survival and land constraints hindered broader adoption. In conclusion, while the study reveals the benefits of AFPs, it emphasizes the need for targeted interventions to address socio-economic barriers and enhance practices. Recommendations include addressing the socio-economic factors that influence AFP adoption, improving extension services, promoting sustainable management of existing AFPs, and conducting further research on tree species compatibility with local agroecologies and effective tree management practices.

## CRediT authorship contribution statement

**Jemal Ali Mohammed:** Writing – review & editing, Writing – original draft, Visualization, Supervision, Software, Methodology, Investigation, Data curation. **Zinet Alye Yimam:** Writing – review & editing, Software, Investigation, Data curation, Conceptualization.

## Consent statement

All participants of the study area were informed that consent to participate in the study and publish their data would be assumed on completion and submission of the study questionnaire/survey.

## Ethics approval

This study was reviewed and approved by the evaluation committee of Bahir Dar University with the approval number: 01/1356/115, dated 3, May 2024.

## Data availability

The data used for this study is available from the corresponding author on reasonable request.

## Declaration of competing interest

The authors declare that they have no known competing financial interests or personal relationships that could have appeared to influence the work reported in this paper.
