# Supplementary material for: Household level determinants of agroforestry practices adoption in rural Legambo district of northcentral Ethiopia
Source: Heliyon. 2025 Feb 18;11(4):e42765. doi: 10.1016/j.heliyon.2025.e42765 (PMC11909435; doi:10.1016/j.heliyon.2025.e42765)
Supplement: Multimedia component 1 [file mmc1.docx]

# **Supplementary Materials**

**Appendix 1:** Research Questionnaires

**Part 1: Questionnaires prepared for face-to-face interview with sample HHs**

- 1. Gender of HH head/respondent.

| (1) Male [ ] | (2) Female [ ] |
| --- | --- |

- 1. Age of HH head/respondent.

| (1) Below 30 [ ]  (2) 30–45 [ ] | (3) 46–64 [ ]  (4) 65 and above [ ] |
| --- | --- |

- 1. Education level of the HHs

| (1) No education [ ]  (2) 5-8 school [ ]  (3) 1-4 school [ ] | (4) 9-12 school [ ]  (5) Certificate/diploma [ ] |
| --- | --- |

- 1. HH size.

| (1) 1–3 [ ]  (2) 4–6 [ ] | (3) 7–9 [ ]  (4) Above 9 [ ] |
| --- | --- |

- 1. Do you have a farm land?

| (1) Yes [ ] | (2) No [ ] |
| --- | --- |

1. How did you obtain your farm land?

| (1) Lease [ ]  (2) Freehold [ ] | (3) Given by family [ ]  (4) Others (Specify) [ ]…………. |
| --- | --- |

- 1. Total land size available for farming including grazing land (ha)?

| (1) 0.125–0.5 ha. [ ] (2) 0. 51–0.75 ha. [ ] | (3) 0.76–1 ha. [ ]  (4) Over 1 ha. [ ] |
| --- | --- |

1. Did you have contact with any extension agent during the 2015 production year?

| (1) Yes [ ] | (2) No [ ] |
| --- | --- |

If yes, specify how many times?............

| (1) 1 – 4 times [ ]  (2) 5 – 8 times [ ] | (3) 9 – 12 times [ ]  (4) >12 times [ ] |
| --- | --- |

1. Are you a member of an organizations/committees/and political parties?

| (1) Yes [ ] | (2) No [ ] |
| --- | --- |

- 1. Do you engage in other activities apart from farming?

| (1) Yes [ ] | (2) No [ ] |
| --- | --- |

- 1. Did you face any land conflicts in the last 12 months with your neighbor land holder?

| (1) Yes [ ] | (2) No [ ] |
| --- | --- |

- 1. Do you own livestock?

| (1) Yes [ ] | (2) No [ ] |
| --- | --- |

- 1. State your economic status.

| (1) Poor [ ]  (2) Medium [ ] | (3) Rich [ ] |
| --- | --- |

**Part 2: Information on the different types of AFPs**

- 1. Do you currently practice any of the following AFPs? (Check all that apply):

| (1) Home garden [ ]  (2) Woodlot [ ]  (3) Trees on cropland [ ] | (4) Alley cropping [ ]  (5) Boundary plantation [ ]  (6) Trees on rangelands [ ] |
| --- | --- |

- 1. If yes, for how long have you been practicing these technologies? ___ years.
  2. What is the extent of your practice? (e.g., number of trees, area covered)

| (1) Homegarden [ ]  (2) Woodlot [ ]  (3) Trees on cropland [ ] | (4) Alley cropping [ ]  (5) Boundary plantation [ ]  (6) Trees on rangelands [ ] |
| --- | --- |

**2.4** Do you regularly manage the practices on your land?

| (1) Yes [ ] | (2) No [ ] |
| --- | --- |

**Part 3: Perceptions of farmers regarding the adoption of AFPs (please rate 1-5)**

| №. | Statement | Response | | | | |
| --- | --- | --- | --- | --- | --- | --- |
|  |  | Str. agree | Agree | Neutral | Disagree | Str. disagree |
| 3.1 | Adoption of AFPs conserves soil and water |  |  |  |  |  |
| 3.2 | Adoption of AFPs improves soil nutrients |  |  |  |  |  |
| 3.3 | Adoption of AFPs increases farm income |  |  |  |  |  |
| 3.4 | AFPs can improves soil cover |  |  |  |  |  |
| 3.5 | Adoption of AFPs improves micro-climate |  |  |  |  |  |
| 3.6 | Adoption of AFPs improves crop production |  |  |  |  |  |
| 3.7 | AFP reduces fuel wood gathering time |  |  |  |  |  |
| 3.8 | AFPs practices have an economic advantage |  |  |  |  |  |
| 3.9 | AFPs reduced risk of complete crop failure |  |  |  |  |  |
| 3.10 | Adoption of AFPs conserves soil and water |  |  |  |  |  |

**Part 4: Questionnaires prepared for interview with KIs**

- 1. How do you describe agroecology of your working district? […………………………………………………………………………………………………………………………………………………………..………………………………………………………………………………]
  2. How do you describe AFPs in your kebele?

[……………………………………………………………………………………………………………………………………………...…………...………………………………………………………………………………]

- 1. Is there any AFPs practiced in your area?

| (1) Yes [ ] | (2) No [ ] |
| --- | --- |

If your answer is yes, what are the most adopted AFPs?

[…………………………………………………………………………………...…..…………………………………………...…………………………………………………………………………………………………]

- 1. If your answer is no, what are the reasons?

[…………………………………………………………………………………………………………………………………………………………………………..………………………………………………………………]

- 1. How do you describe the motivation and contact of farmers with agricultural and NRM experts for advice?

[……………………………………………………………………………………………………………………………………………………………………………..……………………………………………………………]

- 1. What is the perception of farmers towards the benefits of AFPs?

[…………………………………………………………………………………………………………………………………………………………………………………………………………………………………………]

- 1. What are the local environmental challenges related with adopting AFPs?

[…………………………………………………………………………………………………………………………………………………………………………………………………………………………………………]

**Part 5: Questionnaires prepared for FGDs**

- 1. What are the local environmental challenges related with adopting AFPs?

[…………………………………………………………………………………………………………………………………………………………………………………………………………………………………………]

- 1. What factors affect smallholder farmers' adoption of AFPs?

[…………………………………………………………………………………………………………………………………………………………………………………………………………………………………………]

- 1. What do farmers in your local area think about AFPs and their benefits?

[…………………………………………………………………………………………………………………………………………………………………………………………………………………………………………]

- 1. What types of AFPs are used by farmers in your local area?

[…………………………………………………………………………………………………………………………………………………………………………………………………………………………………………]

- 1. Do you think adoption of AFPs improved your crop production? If the answer is yes how?

[…………………………………………………………………………………………………………………………………………………………………………………………………………………………………………]

- 1. What are the barriers that hinders the perceptions of farmers towards AFPs?

[…………………………………………………..………………………………………………………………………………………………………………………………………………………………………………………]

**Appendix 2:** Lists of stratified kebeles by AEZs in Legambo district

| №. | Highland AEZs | Midland AEZs |
| --- | --- | --- |
| 1 | Werento | Gerezewiya |
| 2 | Key mebrat | Genetie |
| 3 | Feta | Selamber |
| 4 | Fesho | Wede-hamusye |
| 5 | Buso | Hetetera |
| 6 | Yetinora | Yilada |
| 7 | Gurach | Kore |
| 8 | Dereba | Arigiti |
| 9 | Tincha | Sada Korkora |
| 10 | Chuluke |  |
| 11 | Bwabwato |  |
| 12 | Kindo |  |
| 13 | Tach Akasta |  |
| 14 | Tikile |  |
| 15 | Gol |  |
| 16 | Mesobit |  |


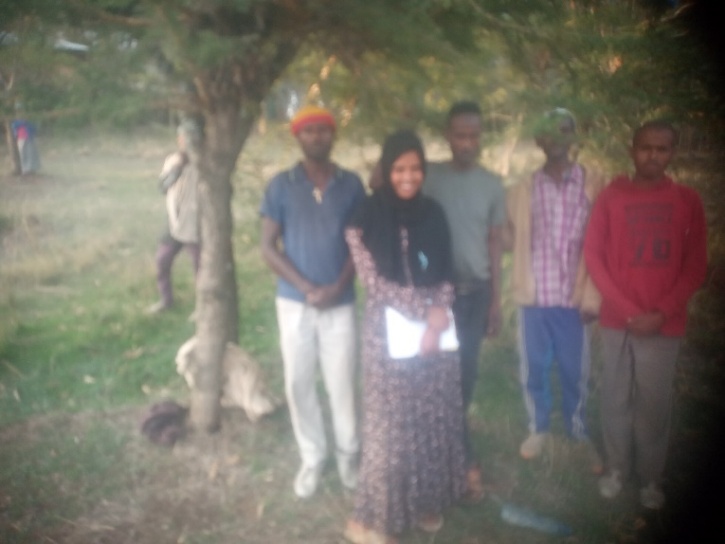

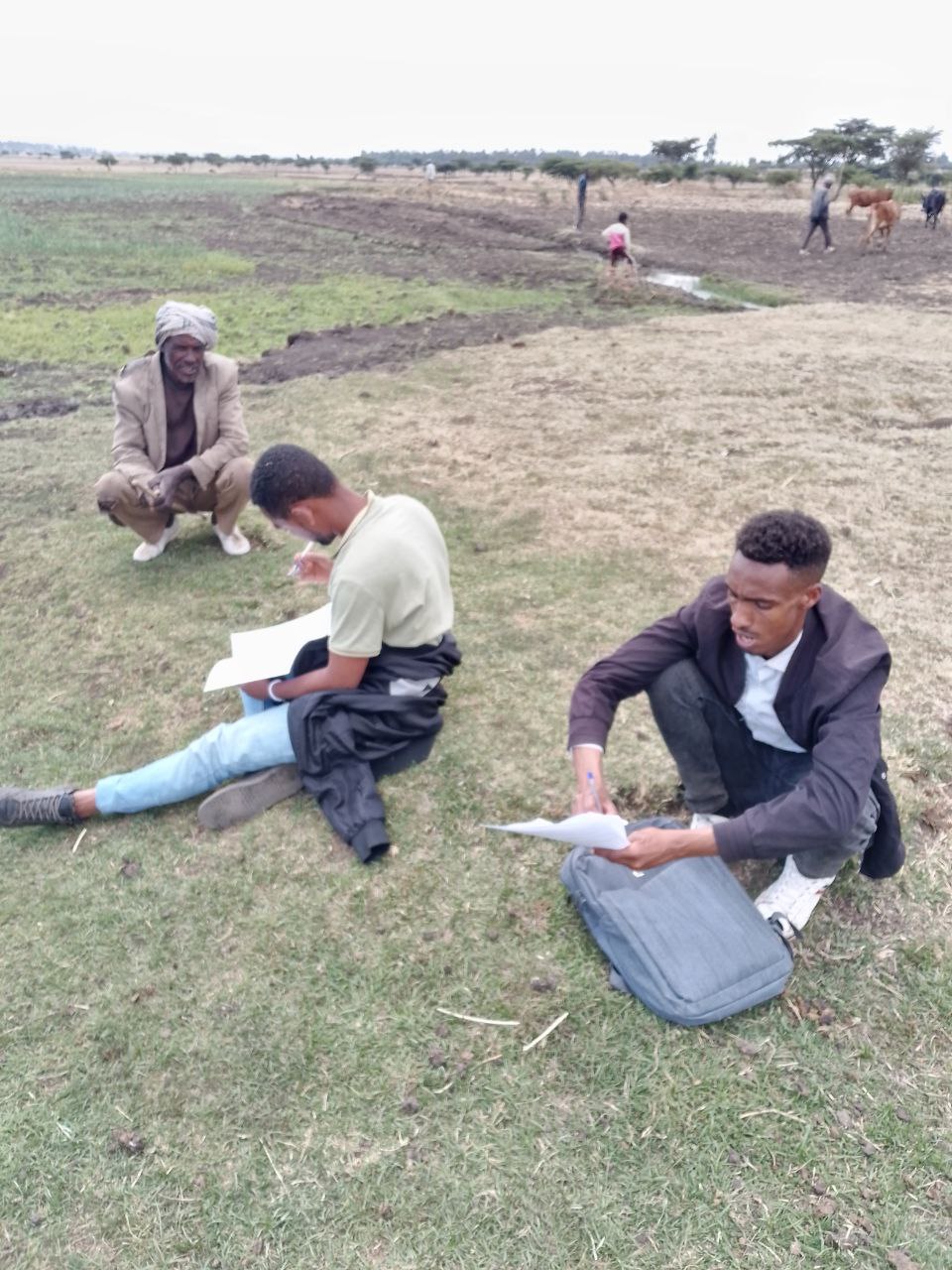


**Appendix 3:** Sample photos taken with respondents during face-to-face interviews and FGDs (source: authors, 2023).


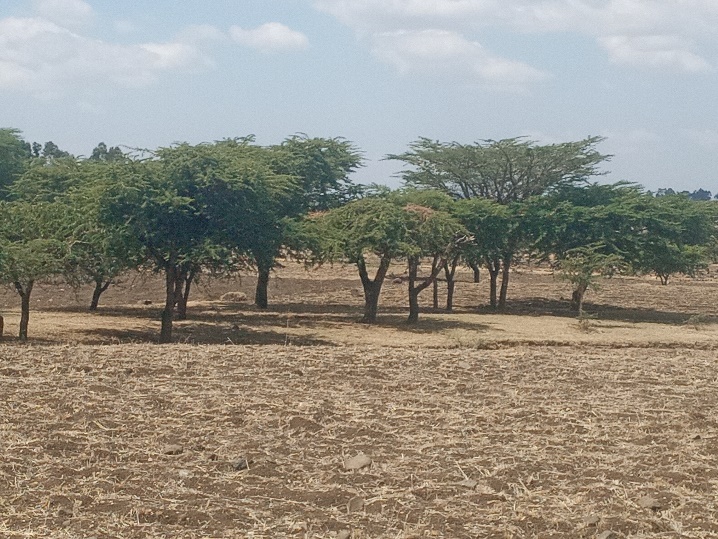

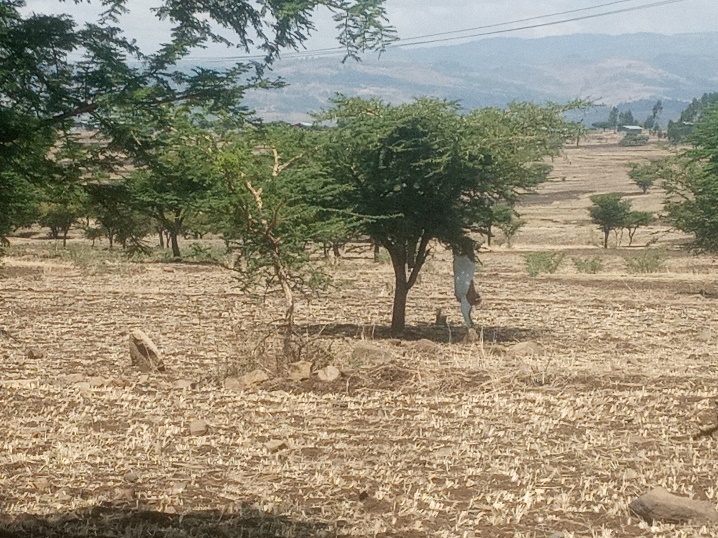


**Scattered trees on cropland**


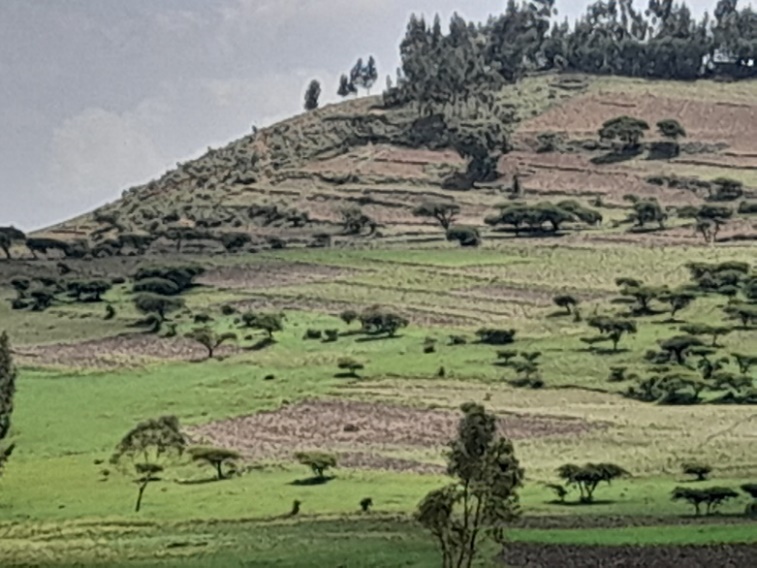

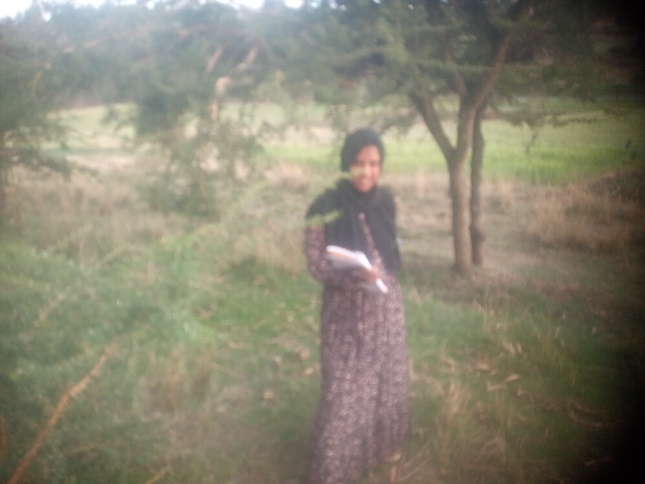


**Scattered trees on range land**


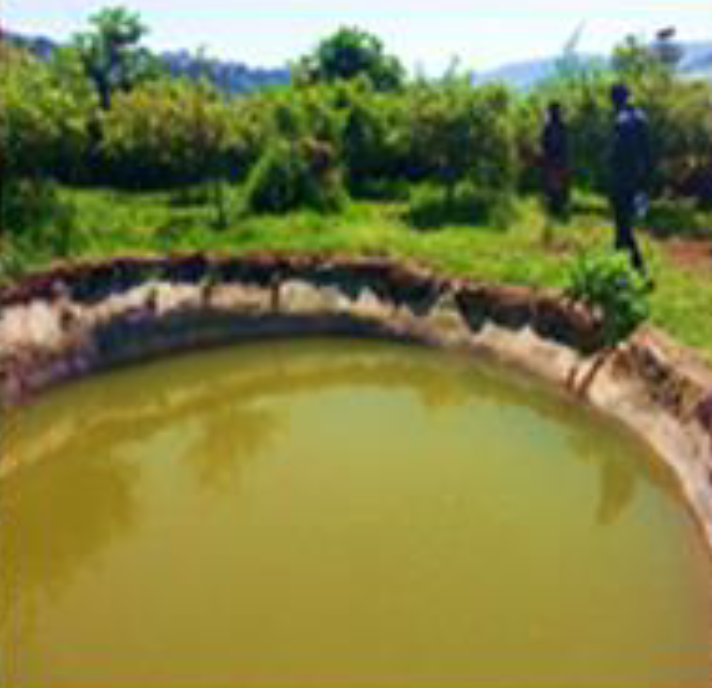

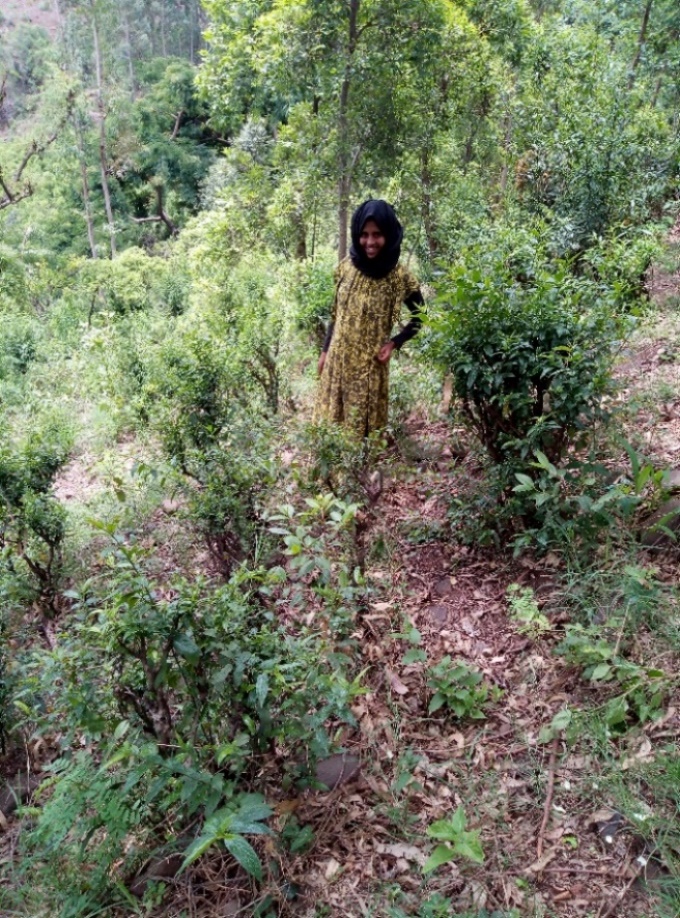


**Home garden**


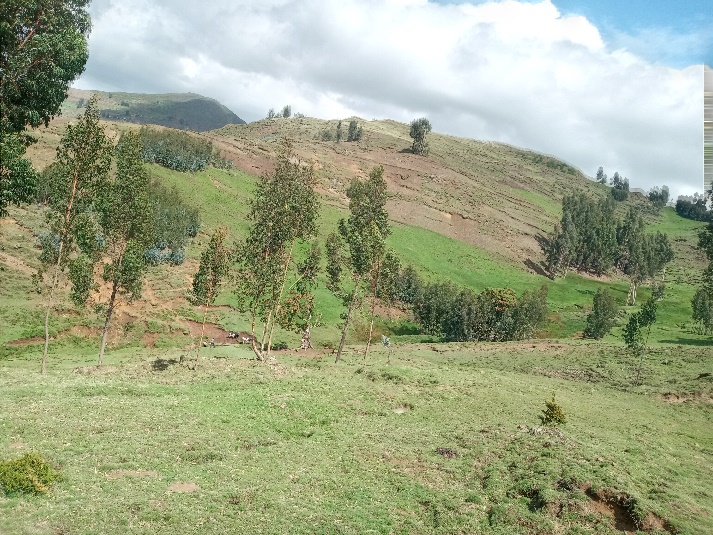

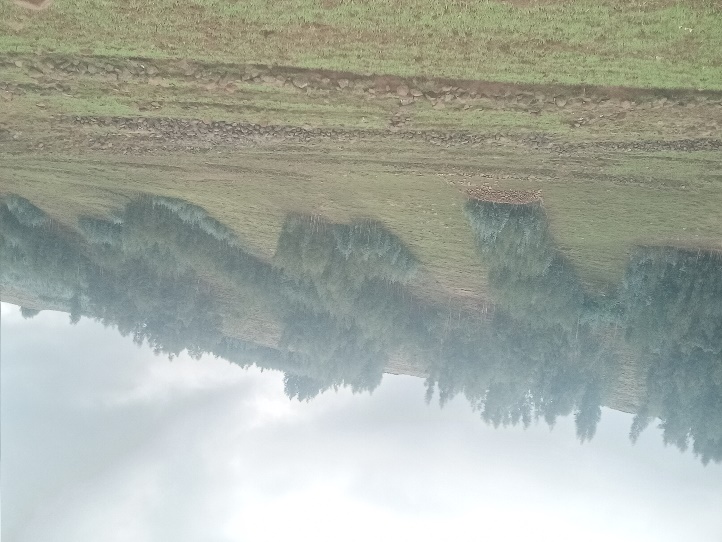


**Multi-purpose woodlots**


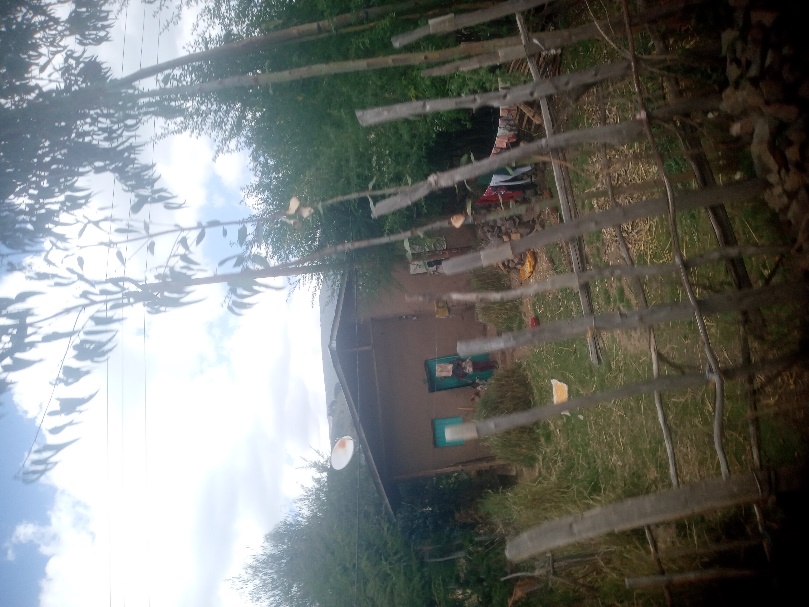

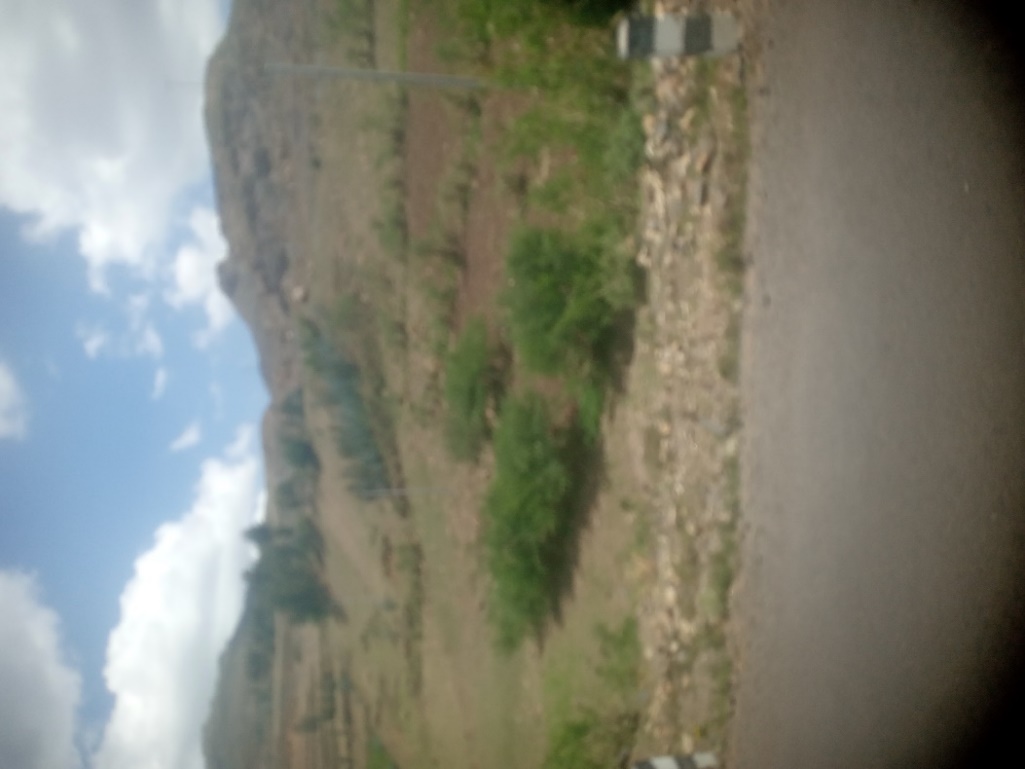


**Alley-cropping**

**Boundary plantation**

**Appendix 4:** Typical AFPs observed during field observation in Legambo district (source: authors, 2023).
